# A functional electrical stimulation system for human walking inspired by reflexive control principles

**DOI:** 10.1177/0954411917693879

**Published:** 2017-03-06

**Authors:** Lin Meng, Bernd Porr, Catherine A Macleod, Henrik Gollee

**Affiliations:** 1 Division of Biomedical Engineering, University of Glasgow, Glasgow, UK; 2 Department of Biomedical Engineering, University of Strathclyde, Glasgow, UK

**Keywords:** Functional electrical stimulation, gait assistance, reflexive mechanism, human walking, rehabilitation

## Abstract

This study presents an innovative multichannel functional electrical stimulation gait-assist system which employs a well-established purely reflexive control algorithm, previously tested in a series of bipedal walking robots. In these robots, ground contact information was used to activate motors in the legs, generating a gait cycle similar to that of humans. Rather than developing a sophisticated closed-loop functional electrical stimulation control strategy for stepping, we have instead utilised our simple reflexive model where muscle activation is induced through transfer functions which translate sensory signals, predominantly ground contact information, into motor actions. The functionality of the functional electrical stimulation system was tested by analysis of the gait function of seven healthy volunteers during functional electrical stimulation–assisted treadmill walking compared to unassisted walking. The results demonstrated that the system was successful in synchronising muscle activation throughout the gait cycle and was able to promote functional hip and ankle movements. Overall, the study demonstrates the potential of human-inspired robotic systems in the design of assistive devices for bipedal walking.

## Introduction

Functional electrical stimulation (FES) has been widely used in rehabilitation strategies for neurologically impaired individuals.^1–6^ The purpose of an FES intervention is to enable functional movement by replacing or assisting with a person’s voluntary muscle activation. Compared to conventional physiotherapy, FES can enhance motor learning and increase central nervous system (CNS) plasticity.^7^

A neural prosthesis based on FES is used to substitute for lost neurological functions. Crucial to the functional effectiveness of an FES system for gait is the correct timing of the applied stimulation within the gait cycle.^8^ The simplest method to control the timing of the stimulation is by manual button press or foot switch and is used in the majority of commercial products. In the 1960s, Liberson et al.^9^ proposed the first portable device for correcting drop foot by stimulating the peroneal nerve in the swing phase, detected via a foot switch. The first commercial FES system for gait, Parastep I, became available in the 1990s.^10^ The open-loop system applies surface stimulation to the quadriceps, gluteal muscles and common peroneal nerve and is controlled through a hand switch integrated into a walking frame. Although open-loop control is a simple and reliable approach to controlling the stimulation, it requires the continuous attention of the operator, and any mistiming of stimulation can result in abnormal muscle synchronisation within gait cycle.

Biologically inspired control with the integration of sensory feedback has been proposed as a promising method for synchronising muscle stimulation to restore functional movement.^11^ In the last decade, locomotion control with a hierarchical structure has become popular in real-time FES gait systems.^12,13^ The top level of the controller determines the stimulation state of muscles, which enables an accurate and automatic synchronisation of multiple muscles. Most systems apply constant stimulation sequences to muscles in the lower level.^14–19^ However, various machine learning approaches have been incorporated with finite state control (FSC) methodology to regulate parameters, such as pulse width or current amplitude, with precise control of kinematic or kinetic data during gait.^20,21^ The use of artificial neural networks (ANN) to create stimulation patterns required for FES gait has also previously been reported.^22^

In contrast to previous approaches, we have investigated the use of a purely reflexive algorithm to generate robust gait patterns. This approach has been inspired by the concept of a ‘passive-dynamic walker’ as implemented in the RunBot bipedal robot, which is driven by local reflexes without any use of position or trajectory tracking control and without using a central pattern generator.^23,24^ The original RunBot used a biologically inspired neural network controller where motor outputs were generated by ground contact inputs with the help of a spiking neural network.^23^ However, the locomotion control in the CNS is highly complicated with numerous unknown variables. In order to avoid the problems associated with a multitude of uncertain biological parameters, we decided to investigate whether the relationship between foot contact and muscle activation in human walking can be described by linear transfer functions.^25^ The transfer functions were derived from leg muscle activity and foot contact data recorded from healthy subjects during treadmill walking and mapped onto the robotic control strategy of a bipedal robotic walker (RunBot II). The results showed that our black box approach enables us to model the complex neural control system in humans and shows how input signals can be translated into functional motor outputs.

The study presented here demonstrates a novel multichannel FES gait system based on a purely reflexive mechanism which is aimed at assisting gait locomotion in patients with walking impairments. As described above, the stimulation strategy utilises transfer functions extracted from healthy subjects where the transfer functions translate foot contact inputs into muscle activation outputs. The article is structured as follows. We first describe the gait phase detection algorithm and the principles of the stimulation strategy and propose a multichannel FES gait system. The results from FES-assisted treadmill walking using healthy subjects are then presented. Gait kinematics were analysed and compared between conditions of normal and FES-assisted treadmill walking to demonstrate the functionality of the system.

## Materials and methods

**Figure 1:**
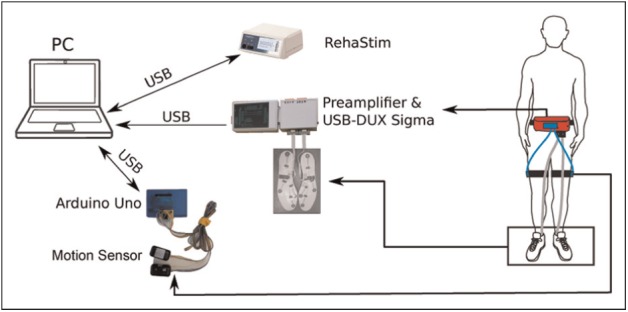
The structure of the FES system. The system consists of four main parts, a programmable stimulator (RehaStim), data acquisition devices (USB-DUX Sigma and Arduino Uno), sensors and a host computer. The participant wears the data acquisition devices around the waist, the FSR embedded insoles are placed in the shoes and motor tracking sensors are positioned on the lateral side of the thighs.

The RunBot III is the basis of our black box controller and is the next generation of the RunBot II,^25^ where the control of ankle movement during the gait cycle has been integrated for the first time. The prototype FES-assisted gait training system features a sensor system to provide sensory input which consists of force sensitive resistors (FSRs) embedded into shoe insoles and two miniature inertial measurement units (IMUs). The FSRs (FSR 402; Interlink Electronics Inc., USA) are used to measure the ground reaction force during walking. As shown in Figure 1, the positions of FSRs under the foot are underneath the heel, first metatarsal head, fifth metatarsal head and the big toe. Note here, the FSR signal of the toe was excluded from the final control system due to its inter- and intra-subject variation.

The insoles were custom-made to the various shoe sizes of the participants. 9-axis MotionTracking MEMS (microelectromechanical systems) devices (MPU9150; InvenSense, USA) consist of accelerometers and gyroscopes measuring angular rate and acceleration about three orthogonal axes. Hip sagittal angular position is computed through a complementary filter algorithm.^26^

The FSR signals are pre-amplified with gain of 1000 before sampling. All sensory signals are sampled with a frequency of 100 Hz and transferred onto a host laptop through Universal Serial Bus (USB) ports. The USB-DUX Sigma (Incite Technology Ltd, UK) and Arduino Uno are used as data acquisition devices for FSR and hip sagittal angle signals, respectively.

The RehaStim system (RehaStim 2; HASOMED, Germany) has eight surface stimulation channels on two separately controlled modules designed to deliver overlapping pulse trains for producing complex movement patterns. The stimulator is connected through an USB port and is controlled by a protocol called ScienceMode.

The algorithms described in the following sections have been implemented in a C++ program and run on a laptop using the Linux operating system. A graphical user interface (GUI) was created to allow customisation of the stimulation protocol and monitor the training.

### Gait phase detection

A gait phase detection algorithm has been developed where one gait cycle is divided into five gait phases, namely, the loading response, stance, pre-swing, swing and terminal swing. An IF-THEN type finite state machine is employed in this system. The state machine is similar to that described by Pappas et al.^27^ However, we utilised a combination of IMUs and FSRs allowing to detect the swing and terminal swing phases which were not integrated in previous system.^8,27^

**Figure 2:**
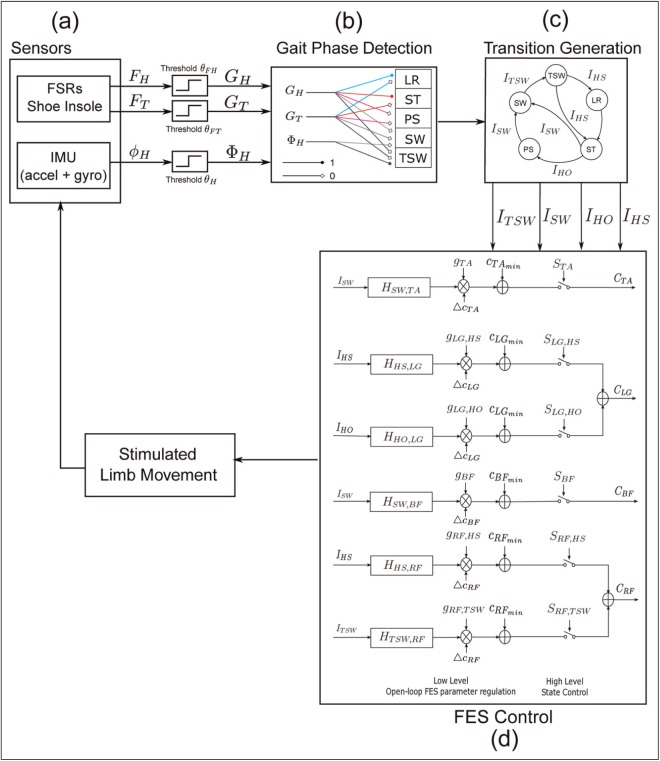
The FES system diagram. (a) The foot contact signals are measured by shoe insoles embedded with force sensitive resistors (FSRs), while the sagittal hip angular signal is computed from the accelerometer and gyroscope. The signals (heel contact $F_{H}$, toe contact $F_{T}$ and hip angle $\phi_{H}$) are translated into binary signals by an adaptive threshold method. (b) Gait phases are detected based on sensory inputs and setup rules. (c) Event impulses are generated when transitions between gait phases occur. (d) A hierarchical FES control model consisting of two levels of control. The top level control switches the stimulation of muscles on and off controlled by event trigger impulses. The stimulation current intensity is regulated in the low level of FES control. LR: load response; ST: stance; PS: pre-swing; SW: swing; TSW: terminal swing.

In our case, the sensor signals to the finite state machine include FSR signals ($F_{H}$ and $F_{T}$) and the hip angle in the sagittal plane ($\phi_{H}$) as shown in Figure 2(a). An adaptive threshold method is used to convert the inputs to binary signals. $G_{H}$ is a binary signal from the FSR signal of the heel ($F_{H}$). $F_{T}$ is the maximal value of two FSR signals from under the first and fifth metatarsal head since the foot load is usually not symmetrical. $G_{T}$ is a binary signal from the FSR signal of the forefoot ($F_{T}$). The logic value 1 indicates that the specific part of the foot is in contact with the ground and 0 indicates that the segments are lifted off the ground. $\Phi_{H}$ is a binary signal from the sagittal hip angle. It is used to determine the terminal swing phase when the foot is lifted off the ground ($G_{H}=0$, $G_{T}=0$). $\theta_{\mathrm{FH}/\mathrm{FT}/H}$ are the threshold values.

Event impulses are generated during transitions between states as shown in Figure 2(c). Four types of impulses are required for the FES control, $I_{\mathrm{HS}}$, $I_{\mathrm{HO}}$, $I_{\mathrm{SW}}$ and $I_{\mathrm{TSW}}$. A summary of the rules generating these impulses is given below:

$I_{HS}$: The impulse indicates the initial foot contact with the ground. In normal gait, the heel usually strikes the ground first. However, individuals with a pathological walk may establish the foot contact with the forefoot. Therefore, the transition is detected if any part of foot touches the ground after the swing phase
($G_{H}(t)=1$ or $G_{T}(t)=1$) and ($G_{H}(t-1)=0$ or $G_{T}(t-1)=0$)
$I_{\mathrm{HO}}$: The transition occurs when the FSR underneath the heel is not pressed and the forefoot is still in contact with the ground. This event indicates a transition from the stance phase to the pre-swing phase
($G_{H}(t)=0$ and $G_{T}(t)=1$) and ($G_{H}(t-1)=1$ and $G_{T}(t-1)=1$)
$I_{\mathrm{SW}}$: The impulse indicates the transition from the stance or pre-swing phase to the swing phase, where the swing phase is when the foot is lifted entirely off the ground so that no FSRs are pressed
($G_{H}(t)=0$ and $G_{T}(t)=0$) and ($G_{T}(t-1)=1$)
$I_{\mathrm{TSW}}$: The impulse indicates the transition from the swing phase to terminal swing phase when the hip flexes forward and the measured $\phi_{H}$ reaches its threshold
($G_{H}(t)=0$ and $G_{T}(t)=0$) and ($\Phi_{H}(t)=1$ and $\Phi_{H}(t-1)=0$)

### Stimulation strategy

After event impulses are detected by the gait detection system, they are fed into the control algorithm for the generation of stimulation sequences. Four muscles were selected for activation, namely, the tibialis anterior (TA), lateral gastrocnemius (LG), biceps femoris (BF) and rectus femoris (RF) as these are muscles associated with different flexion/extension functions during walking and were the focus of previous research.^25^ A hierarchical controller was created based on the robotic reflexive controller as shown in Figure 2(d).

The top level implements an FSC model where the state function *S* switches on and off electrical stimulations of muscles, thereby timing and coordinating the muscle activations (equation (1))

(1) $$\begin{array}{l} S_{TA}=\{\begin{array}{cc} 1 & \mathrm{state}=\mathrm{swing}/\mathrm{terminate swing}\\ 0 & \mathrm{otherwise} \end{array}\\ S_{BF}=\{\begin{array}{cc} 1 & \mathrm{state}=\mathrm{swing}/\mathrm{terminate swing}\\ 0 & \mathrm{otherwise} \end{array}\\ S_{LG,\;HS}=\{\begin{array}{cc} 1 & \mathrm{state}=\mathrm{loading response}\\ 0 & \mathrm{otherwise} \end{array}\\ S_{LG,\;HO}=\{\begin{array}{cc} 1 & \mathrm{state}=\mathrm{pre – swing}\\ 0 & \mathrm{otherwise} \end{array}\\ S_{RF,\;HS}=\{\begin{array}{cc} 1 & \mathrm{state}=\mathrm{loading response}\\ 0 & \mathrm{otherwise} \end{array}\\ S_{RF,\;TSW}=\{\begin{array}{cc} 1 & \mathrm{state}=\mathrm{terminate swing}\\ 0 & \mathrm{otherwise} \end{array} \end{array}$$

The stimulation amplitude is adjusted by convolving an event impulse (i.e. transition between finite states) with a transfer function *H* in the lower level part of controller. These transfer functions were estimated in our previous study^25^ where finite impulse response (FIR) filter coefficients were calculated via an iterative optimisation algorithm based on the FSR inputs and electromyograph (EMG) outputs collected from healthy volunteers during treadmill walking. We then turned these impulse responses into second-order low-pass Butterworth filters via curve fitting. This strategy produces computationally efficient functions which are suitable for real-time implementation. The profiles of the impulse responses are mainly determined by their cut-off frequencies $f_{c}$. The parameter $f_{c}$ for each transfer function is related to the phase duration when the muscle is activated. Equation (2) shows the mathematical expression of the generation of the response output

(2) $$\begin{array}{c} U=g\cdot H*I \end{array}$$

where *H* is the transfer function which is convolved with the impulse input *I* to generate the response output. *g* is the gain coefficient to normalise the response output to a range between 0 and 1.

By assuming a relationship between a specific stimulation channel and one movement, it is possible to generate gait patterns by varying the stimulation parameters on a gait cycle basis. The stimulation frequency is fixed at 40 Hz. The pulse width is set to 350 μs, individually for each muscle, and the current amplitude is updated corresponding to the response output that is regulated to a range between the minimum threshold $c_{min}$ and the maximum threshold $c_{max}$ so that an output of *G* in equation (2) of zero corresponds to $c_{min}$ and the maximum value of *G* to $c_{max}$. The switch function *S* sets the stimulation of each muscle to zero when the pre-set states are not detected. The generation of stimulation sequences for individual muscles can be expressed as follows

(3) $$\begin{array}{c} C_{TA}=(U_{TA}\cdot \Delta c_{TA}+c_{TA_{min}})\cdot S_{TA}\\ C_{LG}=(U_{LG,HS}\cdot \Delta c_{LG}+c_{LG_{min}})\cdot S_{LG,HS}\\ +(U_{LG,HO}\cdot \Delta c_{LG}+c_{LG_{min}})\cdot S_{LG,HO}\\ C_{BF}=(U_{BF}\cdot \Delta c_{BF}+c_{BF_{min}})\cdot S_{BF}\\ C_{RF}=(U_{RF,HS}\cdot \Delta c_{RF}+c_{RF_{min}})\cdot S_{RF,HS}\\ +(U_{RF,TSW}\cdot \Delta c_{RF}+c_{RF_{min}})\cdot S_{RF,TSW} \end{array}$$

where *U* is the response output of transfer function. Δc is the difference between $c_{min}$ and $c_{max}$. $c_{max}$ is the maximum threshold current that can produce a maximal muscle contraction, and $c_{min}$ is the minimum threshold current that can elicit a muscle contraction which can be visually observed. The values of $c_{max}$ and $c_{min}$ for each muscle were measured during a preparation trial prior to the treadmill walking. The state functions *S*, which switch the stimulation on and off, were defined in equation (1).

## System testing

Tests were conducted to evaluate the reliability and repeatability of the FES gait assistive system. The behaviour of the system was evaluated with healthy volunteers. The performance of treadmill walking when stimulation was applied to the muscles was compared to normal treadmill walking without stimulation.

### Ethics statement and participants

The College of Science and Engineering Ethics Committee, University of Glasgow approved the protocol. Seven healthy individuals (five males and two females) with no known gait impairments participated in the study. The mean (standard deviation (SD)) age was 28.7 (7.9) years and the mean (SD) height was 1.75 (0.08) m. The participants were fully informed of the testing procedure and provided written consent prior to the study starting.

### FES setup

Four leg muscles were stimulated in the study: RF, BF, LG and TA of both legs, in order to augment knee flexion/extension and ankle flexion/extension. Stimulation of the RF and BF aimed to induce hip flexion/extension. All electrodes were carefully placed at the appropriate anatomical locations to produce sufficient muscle contraction of the desired muscles. The frequency of the stimulation was set to 40 Hz, and the pulse width was 350 μs. The current stimulation sequence was generated as described in equation (3).

Prior to the FES treadmill walking session, a preparation session was conducted for each participant where the stimulation current parameters were tested so the minimal threshold current $c_{min}$ and maximal threshold current $c_{max}$ could be set in the FES system. These parameters were determined for each muscle in turn by increasing the electrical current amplitude incrementally from 0 mA in steps of 2 mA. The researcher determined the values of $c_{min}$ and $c_{max}$ by observation of the muscle contractions. The setup parameter values are detailed in Table 1.

**Table 1:** Stimulation parameters determined in the FES setup. Four muscles of each leg were chosen in the study, namely, the TA, LG, BF and RF. The parameters $c_{min}$ and $c_{max}$ were measured for each muscle. The units are mA.

Subjects		LTA	LLG	LBF	LRF	RTA	RLG	RBF	RRF
A	$c_{min}$	10	16	14	14	12	12	18	14
	$c_{max}$	20	22	26	20	18	14	22	22
B	$c_{min}$	6	14	8	12	10	12	10	14
	$c_{max}$	24	28	22	22	22	24	22	24
C	$c_{min}$	8	8	10	10	6	6	10	10
	$c_{max}$	12	12	14	16	10	10	14	14
D	$c_{min}$	10	10	16	6	10	14	14	18
	$c_{max}$	14	14	22	18	22	22	24	26
E	$c_{min}$	8	8	14	14	8	8	14	14
	$c_{max}$	20	20	22	22	14	26	24	24
F	$c_{min}$	10	12	16	18	10	16	24	16
	$c_{max}$	24	24	26	30	24	26	30	30
G	$c_{min}$	12	6	14	12	14	10	10	10
	$c_{max}$	22	20	24	28	24	24	24	28

LTA: left tibialis anterior; LLG: left lateral gastrocnemius; LBF: left biceps femoris; LRF: left rectus femoris; RTA: right tibialis anterior; RLG: right lateral gastrocnemius; RBF: right biceps femoris; RRF: right rectus femoris.

**Figure 3:**
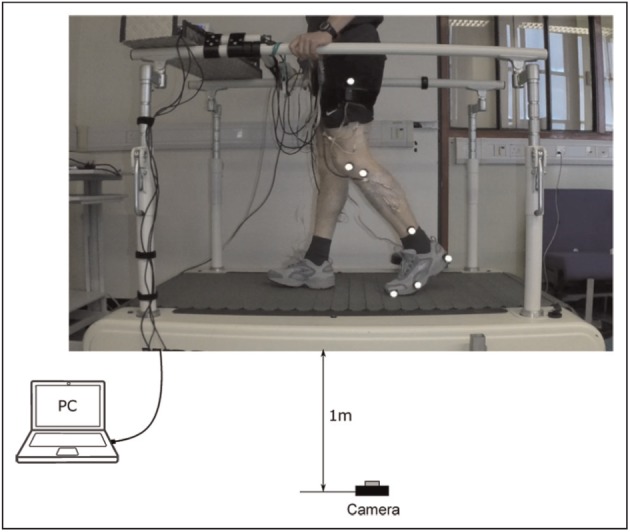
Schematic of the experimental setup: participant walking on the treadmill during muscle stimulation. All devices including the stimulator and data acquisition devices were connected to a PC which runs the control program, while the subject wore the FSR insoles in their shoes and motion tracking MPU9150 on the lateral side of the thigh. A high-speed video camera was used to capture the kinematic motion by tracking retro-reflective markers placed on the lower limb. The ground contact signals from the FSRs, the sagittal plane hip angles computed by the Arduino Uno and stimulation current amplitude for each muscle were also recorded.

Participants were required to wear flat-soled training shoes and shorts. The FSR insoles were placed in the shoes, motion tracking devices were placed on the lateral side of each thigh and the data acquisition devices were worn around their waists. A single camera motion capture system was used to capture the two-dimensional (2D) motion of the left leg in the sagittal plane. The retro-reflective markers were placed on the toe, fifth metatarsal head, heel, lateral malleolus, tibia lateral condyle, femoral lateral epicondyle and greater trochanter of the left leg. The ankle, knee and hip joints were obtained from the optical system. The whole setup of the experiment is shown in Figure 3.

### Procedure

The system testing was conducted in the Centre of Rehabilitation Engineering Laboratory at the University of Glasgow. Participants were instructed to walk on the treadmill (Woodway, USA) at a self-selected comfortable speed. Each subject was instructed to (1) walk normally on the treadmill at their self-selected speed for 3 min; (2) walk on the treadmill for 1 min with electrical stimulation applied to all eight muscles of both legs at the same speed as in session 1, where $c_{max}$ and $c_{min}$ for each muscle were set to the values measured during the preparation session. Participants were also asked to complete a questionnaire to gain feedback on their impression on using the FES system.

### Data analysis

All kinematic data including the hip, knee and ankle angles were obtained from the motion capture system and initially synchronised with the other recorded data, for example, FSR signals. For each trial, a total of 30 gait cycles were extracted from the data sequence. One gait cycle was considered as the interval between consecutive heel strikes of the left foot. The heel strikes were detected by the gait phase detection system. Each gait cycle was re-sampled and time-normalized to 0%–100% with 101 samples.

The range of movement (RoM), maximum and minimum of the hip, knee and ankle were also calculated from the kinematic data in each trial. These values were used to evaluate the differences in gait kinematics between two walking conditions for each participant. Statistical significance was determined using a two-sample *t*-test, with a significance level of 0.05 (MATLAB2014a, The MathWorks, USA). To reduce the likelihood of incorrectly rejecting the null hypothesis (type I error), the level of significance was corrected for the number of comparisons.^28^ Therefore, the critical *p* value was set to p<0.004.

## Results

**Figure 4:**
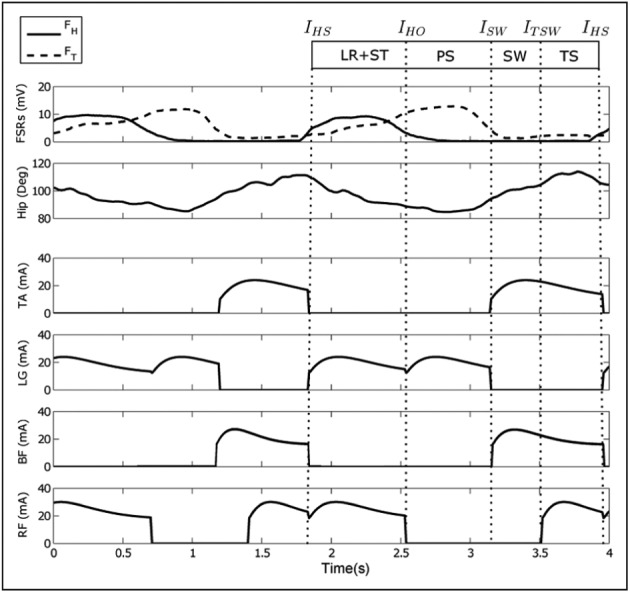
A sequence of 4 s showing consecutive strides recorded during an FES session in one participant. The top two plots show the real-time processed signals from the FSRs and motion sensor. The bottom four plots show the stimulation sequences for four muscles based on the FES controller.

The participants who enrolled in the study walked at a mean (SD) speed of 1.77 (0.25) km/h. The gait event detection system correctly segmented the gait cycle and generated the event impulses. An example of the stimulation sequences and real-time processed signals from the FSRs and motion sensor are provided in Figure 4, for one participant walking with stimulation at his or her self-comfortable speed. The FES strategy was correctly mapped to the duration of gait phases.

**Figure 5:**
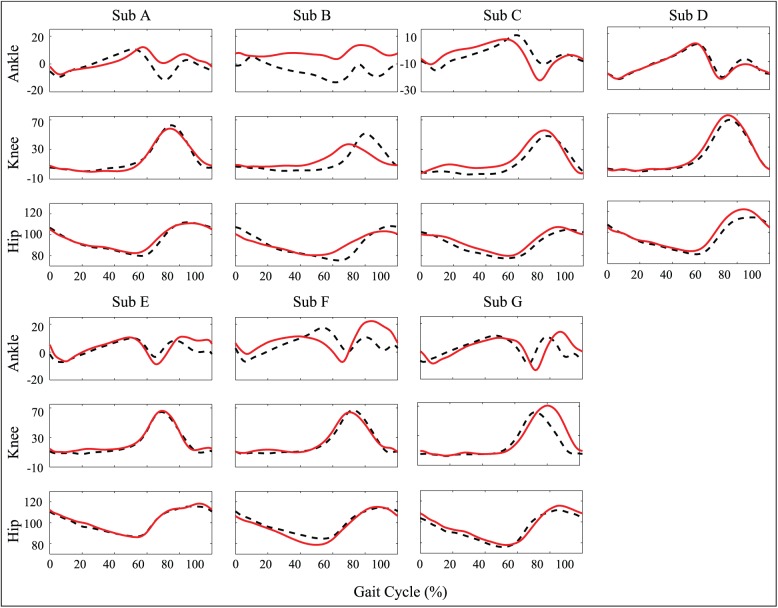
Comparing kinematic data of ankle, knee and hip in each condition (no stimulation vs stimulation). Black dashed lines represent the average joint curves in treadmill walking without stimulation, while red lines show the average joint curves in treadmill walking with stimulation.

**Figure 6:**
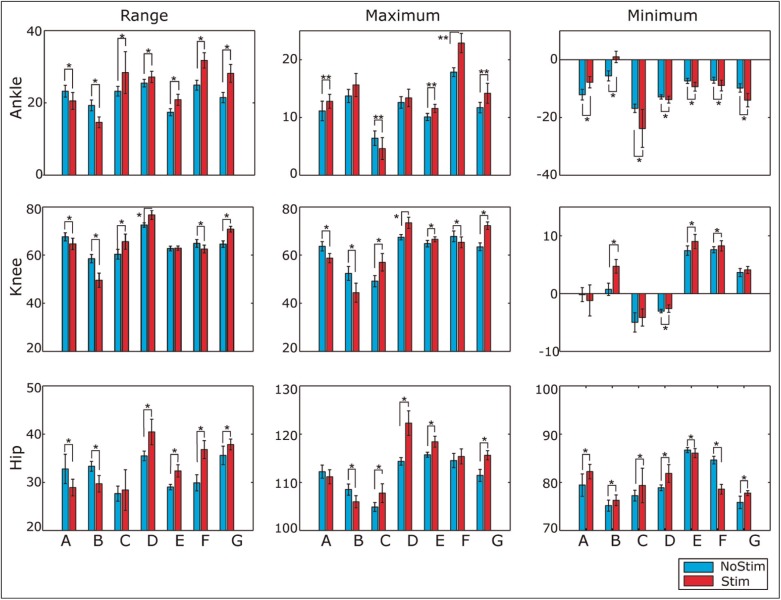
Comparison of kinematic parameters in both conditions. Two-sample *t*-tests were used to evaluate the significant difference between the conditions. *p<0.004, **$p<1e^{-4}$.

All participants achieved a gait pattern with FES similar to their voluntary treadmill gait, as shown in Figure 5, which indicates the FES does not have a negative effect on the gait pattern. Moreover, differences in the joint movement were also noted in Figure 5. The two-sample *t*-test results, as shown in Figure 6, show that the FES has a significant effect on kinematics.

As shown in Figure 5, five of the seven participants obtained a higher peak of ankle plantarflexion angle when the stimulation was applied to the LG muscle during pre-swing phase. Five of the seven participants achieved a larger angle of ankle dorsiflexion in swing phase due to the stimulation applied to the TA muscle. Five of seven participants had a wider range of ankle movement when the LG and TA muscles were stimulated. The FES strategy had a significant effect on the ankle movements of all participants, as shown in Figure 6.

The knee extension in the stance phase during stimulated walking was found to be less than the normal knee extension in six of seven participants. Only four of seven obtained a greater knee flexion angle in the swing phase under the condition of FES. However, an earlier knee extension in the terminal swing phase was observed in six of seven participants, as shown in Figure 5. Quantitatively, the majority of the knee parameters in all participants were significantly different between the two trials, see Figure 6.

When comparing hip joint kinematics, all of the measured parameters relating to the hip joint were found to be significantly different between normal and stimulated treadmill walking. This demonstrated that the induced functions of the BF and RF muscles have a significant effect on the hip movement. A wider RoM of the hip during the gait cycle was achieved by five of seven participants, while these individuals performed significantly larger hip flexion in the swing phase due to the stimulation on the BF. Six of seven participants demonstrated less hip extension during the stance phase. This was found to be the result of the FES, accelerating the transition from the stance phase to swing phase.

None of the participants reported any discomfort or issues related to their treadmill walking while using the FES system.

## Discussion

Human walking is a complex task involving an interaction between the nervous and biomechanical systems to produce coordinated muscle activations to develop a functional gait. In the locomotion of humans and animals, the integration of various reflexes contributes to the control of the limbs and regulation of the gait cycle.^29^ Muscle activity is a combined effect of all the synaptic inputs to the motor neurons.^30^ Studying the relationship between muscle EMG and sensory feedback is thus beneficial to gain a better understanding of the neural mechanism for locomotion control. In a previous study,^25^ we investigated the causal relationship between foot contact information and muscle activation during gait, where the motor output was successfully mapped to biomechanical tasks during gait events. The resulting controller was then applied to a mechanical bipedal robotic walker (RunBot II). In this article, our novel reflexive control system was the basis for the development of an FES controller and multichannel system protocol aimed to assist stepping and promote walking in individuals with limited locomotion ability.

The purpose of FES is to compensate for neuromotor pathologies by functioning as a neural prostheses. For gait generation, the FES is applied to nerves which innervate leg muscles with particular motor functions during the swing and stance phases. A reflexive controller based on human data has implications in FES control as providing sensory feedback from the patients should allow a modulation of the stepping and promote limit cycle walking. Our reflexive controller uses a filter which translates the input of foot contact into a motor control signal. As the filter functions are based on the transfer functions derived from the foot contact and muscle activations in human data, the muscle activity output could be mapped to the biomechanical subtasks on the lower limb main muscles.^3^

The use of inertial sensors including gyroscopes and accelerometers within a closed-loop control system has been reported previously in the literature.^8,14,16,19,31^ The sensory feedback from these sensors is used to detect gait phases and measure kinematics, which can then be employed to adapt the output of the system. Braz et al.^31^ proposed a closed-loop FES gait control system utilising a finite state controller with the help of processed kinematic feedback from four motion sensors placed on the shank and thigh segments. The stimulation of the quadriceps and gluteus and peroneal nerve is controlled during the gait sub-phases determined by the sagittal knee angle signal. Andrews et al.^14^ designed a gait phase detector using a cluster of accelerometers attached to the shank for dividing the stance and swing phases. The exclusive use of motion sensors is challenging because of the low signal-to-noise ratio and the necessity for post-processing, such as using a Kalman filter or non-linear filters such as median filters to obtain a precise estimation of the segment movement. Therefore, the majority of developed systems consist of the combination of foot switches or FSRs and motion sensors. For example, a combined system based on feedback from FSR shoe insoles and gyroscope sensors has been shown to work robustly on different terrains.^8^ Using FSRs positioned under the foot and accelerometers attached to the shank as sensory inputs enables the generation of stimulation sequences for four muscles based on the rules learned from the human data.^19^ This sensor configuration has shown satisfactory results in terms of stability and robustness with respect to external disturbances. We designed a similar setup with FSRs placed underneath the heel, metatarsal heads and motion sensors placed on the thighs.

The hierarchical structure of the controller allows management over the complexity.^32^ The top level of the hierarchy determines the finite states, while the lower level is responsible for dynamics. Compared to the limited selectivity of muscles in open-loop control^9^ and inadequate real-time control in traditional closed-loop systems,^32–34^ FES control with a hierarchical structure has a crucial balance of precise control and practical application in a ‘real world’.^13^ A sensor-driven FES paradigm for hemiparetic patients has previously been proposed based on an IF-THEN rule-based control algorithm.^19^ Here, the rules were created by incorporating artificial feedback from FSRs and accelerometers, and the estimated outputs – muscle EMGs from the nonparetic leg of the patient. The authors found that this method provided timing for muscle activation which was in sync with required voluntary movements. Pappas et al.^8^ combined a gyroscope with FSRs to determine gait events which enabled them to detect the swing phase of gait to trigger the stimulation for drop foot. This study addressed the redundancy, nonlinearity and time variability of the system and falls into the category of FSC.^11^ FSCs can provide an accurate and robust algorithm design (see review in Braz et al.^1^). The main difference between the previously discussed control schemes and our presented study is that our approach uses linear filter/transfer functions to translate the input of the foot contact into a muscle stimulation signal. The use of biologically inspired FES strategies has already shown optimal motor relearning results in other studies.^35,36^ Thus, a reflexive controller with the integration of FSC and biomimetic activation may be a promising approach to obtain an optimal therapeutic effects for gait rehabilitation.

The use of filter functions as an alternative to neuronal processing^12,13^ can provide a simple yet robust FES system. Chia et al.^3^ presented an approach where muscle synergies could be extracted using a non-negative matrix factorisation algorithm. The set of muscle synergies was obtained by averaging in a group of healthy subjects. The biomimetic stimulation strategy was mapped to the gait events detected by a real-time algorithm. The results showed that the stimulation profile could be adapted to the gait events and the subjects’ kinematics.

However, the muscle synergies were not directly related to any sensory feedback. In our study, the use of transfer functions provides a method to relate the sensory feedback with the muscle activation.^25^ The system characteristics make it robust, enabling it to adapt quickly to any changes in the walking environment and in response to disturbances. The set of filter functions only requires two parameters: reducing the computational burden and making it straightforward to implement in practice.

The functionality of our FES gait assistive system was tested in a preliminary study involving seven healthy participants. The current amplitudes were set to not exceed the maximal tolerance of the participants in order to reduce the effect of sensory afferent stimulation to gait. The participants were asked to comply with functional movements induced by FES. None of the participants reported any discomfort or disturbances in walking with the stimulation applied. The results demonstrated that our FES control strategy provides an accurate timing of muscle activation that is synchronised with the required voluntary movements. This can be seen in the universal positive results in gait parameters across the participant group which would not be expected when there is a mismatch between voluntary and stimulated muscle activity.

The performance of the system regarding ankle movement shows the same orthotic effect for drop foot correction and forward propulsion to patients with gait abnormality as described by other clinical research.^37–39^ It was also observed that the flexion of the hip, knee and ankle joints were accelerated by the application of FES during the swing phase, especially in early swing.^17^ Invoking hip flexion in addition to ankle dorsiflexion improves foot clearance and leg swing. Our multichannel FES system shows substantial potential to provide assistance to functional movement, which may have an application in gait rehabilitation of patients with neurological injuries or disease, whose walking ability may be reduced.

Our system requires users still retain some residual motor function as sensory feedback is the prerequisite to generate the stimulation sequences and initiate stepping. In particular, individuals who suffer an impairment of the sensor motor system would benefit from the system. Such conditions could include stroke, multiple sclerosis, incomplete spinal cord injuries, Parkinson’s disease and cerebral palsy. Coordination training assisted by our proposed FES system during rehabilitation may improve in the coordinated components of gait.^40^ The system also has potential to enhance motor learning and promote CNS plasticity.^7^

One of the major limitations of FES is that the stimulated muscles tend to fatigue very rapidly. The exact cause of muscle fatigue is unknown but may be related to an exhaustion of the contractile mechanism.^41^ In terms of patients with neuromuscular paralysis, the problem of fatigue is exacerbated by physiological changes to the muscle due to disuse.^42^ Studies have shown that variations in stimulation frequency, pulse pattern and pulse number have little influence on muscle fatigue.^41,42^ However, Kesar and Binder-Macleod^43^ suggested that intermittent high-frequency stimulation produces maximal isometric performance by minimising muscle fatigue than low-frequency repetitive stimulation on healthy and spinal cord injured subjects. In our study, FES is applied intermittently to muscles in specific phases of the gait cycle, which may help reduce muscle fatigue. However, the prediction and prevention of muscle fatigue is not the main concern of this article as our FES system is an assistive system, where fatigue is less of an issue compared to a full neuroprostheses aimed at providing complete gait function to the patient.

The work outlined in this article demonstrated successfully that a robotic algorithm can be used to establish a limit cycle walking in humans and has potential to support the remaining functions of a damaged nervous system. The results demonstrate the benefits of human robotic interaction to robotic engineering and assistive technology development.
